# Risk factors of diffuse alveolar hemorrhage in Chinese patients with systemic lupus erythematosus

**DOI:** 10.1038/s41598-023-49978-2

**Published:** 2023-12-16

**Authors:** Lishan Xu, Rong Yang, Yingping Cao, Meihua Wang, Xuwei Yang

**Affiliations:** 1 Department of Rheumatology, Fujian Medical University Union Hospital, Fuzhou, China; 2 Follow-Up Center, Fujian Medical University Union Hospital, Fuzhou, China; 3 Department of Laboratory Medicine, Fujian Medical University Union Hospital, Fuzhou, China

**Keywords:** Diseases, Rheumatology, Risk factors

## Abstract

This study aimed to investigate the frequency and features of diffuse alveolar hemorrhage (DAH) in Chinese patients with systemic lupus erythematosus (SLE) and evaluate the association of DAH with the features. A total of 943 patients with SLE were categorized into two groups: 896 patients without DAH and 47 patients with DAH. The demographic data, clinical and laboratory findings, and SLE disease activity index 2000 of all patients were statistically analyzed. The DAH frequency in patients with SLE was 4.98%, and the mortality rate of DAH was 42.55%. The clinical features with statistical differences between the two groups were analyzed by multivariate logistic regression, and the results suggested that shorter disease duration [odds ratio (OR): 0.972, 95% confidence interval (CI) 0.946, 0.998], younger age (OR: 0.867, 95% CI 0.764, 0.984), moderate (OR: 25.949, 95% CI 3.316, 203.065) or severe (OR: 24.904, 95% CI 2.675, 231.859) anemia, abnormally elevated levels of urine protein (OR: 10.839, 95% CI 1.351, 86.938) and serum creatinine (OR: 14.534, 95% CI 5.012, 42.142), interstitial lung disease (OR: 6.569, 95% CI 2.053, 21.021), and infection (OR: 8.890, 95% CI 3.580, 22.077) were independent risk factors for the occurrence of DAH in patients with SLE. Moderate or severe anemia was highly suggestive of DAH.

## Introduction

Diffuse alveolar hemorrhage (DAH) is a life-threatening condition characterized by hemoptysis, hypoxemia, anemia, and transient diffuse alveolar infiltration or consolidation in thoracic radiology, with pathological changes in the disruption of the alveolar capillary basement membrane caused by either inflammation or injury to the arterioles, venules, or capillaries; it leads to widespread extravasation of red blood cells into the pulmonary alveolar spaces^1^. The pathogenesis is not completely understood. The over-activation of neutrophils and inflammatory monocyte infiltration may be the basis for the development of DAH^2,3^. The downregulation of lung miR-146a levels in autoimmune-mediated DAH led to increased expression of tumor necrosis factor receptor-associated factor (TRAF) 6 and interleukin (IL)-8, neutrophil extracellular traps (NETs), and apoptosis formation. Intra-pulmonary miR-146a delivery could suppress DAH by reducing TRAF6, IL-8, NETs, and apoptotic expression^4^. Another study reported that miR-155 enhanced inflammatory responses in the development of induced DAH by targeting multiple negative regulators of the NF-κB signaling pathway^5^. Type I interferon was involved in the pathogenesis of DAH in a murine lupus model. TRAF-family-member-associated NF-κB activator (TANK) ameliorated DAH by inhibiting TRAF6 ubiquitination and mediating the negative regulators of NF-κB signal transduction. In the absence of TANK, DAH was caused by type I interferon rather than IL-6. TANK inhibited the cytoplasmic DNA sensor cyclic GMP-AMP synthase-dependent recognition of cytoplasmic DNA to prevent fatal DAH^6^.

The etiologies of DAH can be broadly divided into immune- and non-immune-mediated causes^1^. DAH is a kind of complication of rheumatoid conditions^7^, and systemic lupus erythematosus (SLE) is among the connective tissue diseases associated with DAH^8^. In general, SLE-associated DAH is considered a serious complication with high mortality. The prevalence of DAH among patients with SLE ranges from 0.2 to 5.4%^9^. Even though DAH symptoms may develop rapidly in hours or over a few days^10^, the clinical manifestations are not specific. Early diagnosis and timely progressive treatment are pivotal for the control of this condition. Due to their atypical clinical manifestations, timely distinguishing DAH from infection and lupus pneumonia is difficult, which may lead to the delay of diagnosis or optimal intervention.

Many studies on SLE-related DAH were conducted due to its rapid disease progression and high mortality. At present, it is still one of the hot spots in SLE research. Early recognition of DAH in patients with SLE is important to initiate appropriate therapy, reduce the mortality rate, and prolong the survival time.

In this study, patients with SLE complicated with DAH at a tertiary-care academic center were retrospectively analyzed to elucidate the frequency and clinical and laboratory features of DAH in Chinese patients with SLE and investigate the association of DAH with the clinical features.

## Patients and methods

The medical records of 943 patients with SLE (112 males and 831 females; mean age of 37.45 ± 15.85 years; range of 7–87 years) at the Department of Rheumatology, Fujian Medical University Union Hospital, a tertiary-care academic center, between January 2013, and May 2022, were collected following the Systemic Lupus International Collaborating Clinics (SLICC) 2012 classification criteria^11^. A total of 47 patients with SLE were categorized as the DAH group on the basis of the following evidence of DAH: (1) acute diffuse lung infiltration on chest high-resolution computed tomography (HRCT) scans, characterized by patchy ground-glass opacities without remarkable interlobular septal thickening; (2) loss of hemoglobin (Hb) by > 1.5 g/dL without evidence of bleeding elsewhere compared with the level before hospitalization, or Hb levels < 10.0 g/dL at the time of the chest HRCT scan; (3) one or more pulmonary symptoms or signs, such as hemoptysis, dyspnea, cough, sputum, and hypoxemia; and (4) hemosiderine-laden macrophages in bronchoalveolar lavage^12^. The other 896 patients with SLE were classified as the non-DAH group.

Patients were excluded if they were complicated with the following conditions: Anti-neutrophil cytoplasmic antibody-associated vasculitis, goodpasture syndrome, overlapping syndrome, congenital heart disease, left heart failure, other lung diseases (such as pulmonary tuberculosis, bronchiectasis, lung abscess, lung embolism, and lung neoplasm), pregnancy, and severe coagulopathy disease.

All patients’ clinical manifestations were examined, and the demographic and clinical characteristics, including disease duration, malar rash, epilepsy, arthritis, oral ulcer, photosensitivity, Raynaud’s phenomenon, serositis, dyspnea, moist rales, fever, alopecia, pulmonary hypertension, interstitial pulmonary disease, complicated infection, and visceral damage, were recorded^13^. Laboratory parameters, such as blood and urine routine, biochemical markers, urine protein, immunoglobulin, and auto-antibodies, were collected. Inflammatory indices, including erythrocyte sedimentation rate (ESR) and C-reactive protein (CRP) level, were obtained. Disease activity was assessed using the SLE disease activity index 2000 (SLEDAI-2K) score^14^. All laboratory data were measured at the Department of Laboratory Medicine, Fujian Medical University Union Hospital. All patients with SLE were screened via chest CT. The three CT instruments were GE Discovery CT 750HD (General Electric Company, Waukesha, USA), GE Revolution CT (General Electric Company, Waukesha, USA), and SOMATOM Definition (Siemens Company, Berlin, Germany).

### Statistical analysis

Data distribution was tested by Shapiro–Wilk test. The results were reported as the mean ± standard deviation, quartile, or number (percentage). Normally distributed data were compared with independent sample t test, and non-normally distributed data or ranked data between the two groups were compared using Mann–Whitney U test. Categorical data were compared using Chi-square test or Fisher exact test. After collinearity diagnosis was conducted, multivariate logistic regression was adopted to report the correlation between the selected variables and DAH in all patients with SLE. For the screened risk factors, the receiver operating characteristic (ROC) curve was drawn, and the area under the curve (AUC) was calculated. Data analysis was performed using SPSS software version 25.0. A difference was considered significant if the two-sided *p* value was less than 0.05.

### Ethical considerations

Due to the retrospective nature of the study, informed consent was waived. The informed consent waiver and the study protocol were approved by Fujian Medical University Union Hospital Ethics Committee (2023KY085). The study was conducted in accordance with the principles of the Declaration of Helsinki.

## Results

### Frequency and attacking time of diffuse alveolar hemorrhage

Among the 943 enrolled patients with SLE, 47 patients who fulfilled the SLICC criteria of SLE and the DAH criteria previously described were recruited as test subjects (DAH group). The frequency of DAH in the SLE patients was 4.98%. The age of the patients at the attacking time of alveolar hemorrhage was (31.77 ± 13.69) years. The average time between SLE diagnosis and the onset of alveolar hemorrhage was (1.86 ± 3.29) years.

### Treatment of diffuse alveolar hemorrhage

All of the 47 patients with SLE-DAH received the cornerstone of therapy (1–2 mg/kg/day methylprednisolone). Among them, 15 patients (31.91%) were treated with pulse intravenous methylprednisolone (1.0 g/day for 3 days), 25 (53.19%) with intravenous cyclophosphamide, 24 (51.06%) with pulse gamma immunoglobulin, 11 (23.40%) with plasmapheresis, five (10.64%) with rituximab, 10 (21.28%) with continuous renal replacement therapy, 16 (34.04%) with mechanical ventilation, and one with extracorporeal membrane oxygenation. In this study, the fatality rate of SLE-associated DAH was 42.55%.

### Clinical features with significant differences

No significant difference in terms of sex was found between the DAH and non-DAH groups. However, statistically significant differences were observed between the two groups in terms of age, SLE disease duration, epilepsy, fever, serositis, oral ulcers, alopecia, pulmonary hypertension, interstitial lung disease, SLEDAI-2K score, anemia, proteinuria, microscopic hematuria, hypoalbuminemia, creatinine, urea nitrogen, lactate dehydrogenase (LDH), ESR, CRP, IgM, IgA, and infection (Tables 1, 2). The other items did not exhibit any difference.

**Table 1:** Demographic and clinical features of patients with systemic lupus erythematosus.

Characteristics	Non-DAH group (n = 896)			DAH group (n = 47)			*p*
	N (%)	Mean ± SD	Quartile	N (%)	Mean ± SD	Quartile	
Age (year)		37.7 ± 15.9	(26.0, 48.8)		31.8 ± 13.7	(22.0, 41.0)	0.014
Sex							
Female	789 (88.1)			42 (89.4)			0.788
SLE duration(year)		3.3 ± 5.2	(0.2, 5.0)		1.9 ± 3.3	(0.1, 2.0)	0.004
Malar rash	279 (31.1)			12 (25.5)			0.417
Epilepsy	15 (1.7)			5 (10.6)			< 0.001
Neuropsychological involvement	84 (9.4)			8 (17.0)			0.142
Fever	301 (33.6)			25 (53.2)			0.006
Arthritis	422 (47.1)			24 (51.1)			0.596
Photosensitivity	113 (12.6)			2 (4.3)			0.088
Serositis	252 (28.1)			32 (68.1)			< 0.001
Xerostomia/xeroma	93 (10.4)			3 (6.4)			0.525
Oral ulcer	100 (11.2)			14 (29.8)			< 0.001
Raynaud’s phenomenon	103 (11.5)			4 (8.5)			0.529
Alopecia	161 (8.0)			15 (31.9)			0.017
Rash	251 (28.0)			15 (31.9)			0.562
Pulmonary hypertension	286 (31.9)			24 (51.1)			0.006
ILD	75 (8.4)			9 (19.2)			0.023
SLEDAI							< 0.001
0–4	303 (33.8)			2 (4.3)			
5–9	328 (36.6)			11 (23.4)			
10–14	154 (17.2)			12 (25.5)			
≥ 15	111 (12.4)			22 (46.8)			
Pathogen infection	299 (33.4)			39 (83.0)			< 0.001

SLE, systemic lupus erythematosus; ILD, interstitial lung disease; SLEDAI, systemic lupus erythematosus disease activity index.

**Table 2:** Laboratory findings of patients with systemic lupus erythematosus.

Characteristics	Non-DAH group (n = 896)			DAH group (n = 47)			*p*
	N (%)	Mean ± SD	Quartile	N (%)	Mean ± SD	Quartile	
Leukopenia	336 (37.5)			11 (23.4)			0.051
Anemia							< 0.001
Mild	284 (31.7)			3 (6.38)			
Moderate	195 (21.8)			31 (66.0)			
Severe	40 (4.5)			11 (23.4)			
Extreme severe	4 (0.5)			1 (2.1)			
Thrombocytopenia	242 (27.0)			17 (36.2)			0.17
Urine protein	500 (55.8)			41 (87.2)			< 0.001
Urine RBC	336 (37.5)			38 (80.9)			< 0.001
24-h urine protein	601 (67.1)			46 (97.9)			< 0.001
Hypoalbuminemia	539 (60.2)			46 (97.9)			< 0.001
Hyperglobulinemia	370 (41.3)			14 (29.8)			0.173
Creatinine (μmol/L)		66.4 ± 55.0	(45.0, 70.0)		190.0 ± 179.9	(55.0, 272.0)	< 0.001
Urea nitrogen		5.7 ± 4.3	(3.4, 6.1)		14.0 ± 9.5	(6.2, 19.9)	< 0.001
LDH (IU/L)		266.3 ± 190.7			423.7 ± 310.6	(257.0, 536.0)	< 0.001
ESR (mm/h)		48.2 ± 41.0	(20.0, 62.0)		51.3 ± 33.6	(21.0, 69.0)	0.015
Elevated CRP	357 (39.8)			30 (63.8)			0.001
IgG (g/L)		17.6 ± 9.8	(12.6, 20.6)		16.5 ± 10.1	(10.3, 20.2)	0.056
IgM (g/L)		1.2 ± 0.7	(0.8, 1.4)		0.9 ± 0.4	(0.6, 1.1)	< 0.001
IgA (g/L)		2.7 ± 1.2	(1.8, 3.3)		2.1 ± 1.1	(1.2, 2.8)	< 0.001
Rheumatoid factor	330 (36.8)			15 (31.9)			0.495
Anti-dsDNA	630 (70.3)			37 (78.7)			0.122
Anti-cardiolipin IgM	59 (6.6)			2 (4.3)			0.742
Anti-cardiolipin IgG	203 (22.7)			12 (25.5)			0.647
Anti-β2GP1	194 (21.7)			7 (14.9)			0.270
Anti-ANCA	54 (6.0)			5 (10.6)			0.335
Anti-U1RNP	459 (51.2)			26 (55.3)			0.638
Anti-SM	311 (34.7)			15 (31.9)			0.251
Anti-SSA	589 (65.7)			33 (70.2)			0.477
Anti-SSB	231 (25.8)			9 (19.2)			0.326
Anti-SCL-70	30 (3.4)			3 (6.4)			0.163
Anti-PM-SCL	47 (5.3)			2 (4.3)			1.000
Anti-JO-1	17 (1.9)			1 (2.1)			0.605
Anti-centromere	33 (3.7)			2 (4.3)			0.365
Anti-PCNA	26 (2.9)			3 (6.4)			0.186
Anti-nucleosome	418 (46.7)			28 (59.6)			0.202
Anti-histone	351 (39.2)			25 (53.2)			0.167
Anti-rRNP	351 (39.2)			17 (36.2)			0.715
Anti-mitochondrial	171 (19.1)			7 (14.9)			0.532

RBC, red blood cell; LDH, lactate dehydrogenase; ESR, erythrocyte sedimentation rate; CRP, C-reactive protein; Ig, immunoglobulin; dsDNA, double-stranded deoxyribonucleic acid; β2GP1, beta-2 glycoprotein 1; ANCA, antineutrophil cytoplasmic antibodies; U1RNP, U1 ribonucleoprotein; SM, Smith; SSA/SSB, anti-Ro/anti-La; Anti-SCL-70, anti-topoisomerase I; PM/SCL, polymyositis-scleroderma; JO-1, histidyl-tRNA synthetase; PCNA, proliferating cell nuclear antigen; RNP, ribonucleoprotein; Elevated CRP, CRP > 8 mg/L; Mild anemia, hemoglobin level (g/L) 90–110; Moderate anemia, hemoglobin level (g/L) 60–89; Severe anemia, hemoglobin level (g/L) 30–59; Extreme severe anemia, hemoglobin level (g/L) < 30.

### Single-factor and multivariate unconditional logistic regression analyses

Single-factor unconditional logistic regression was applied to determine the correlation between DAH and all the factors involved. Among the items, age, SLE disease duration, epilepsy, fever, serositis, oral ulcers, alopecia, anemia, interstitial lung disease, pulmonary hypertension, IgM, IgA, SLEDAI-2K score, infection, hypoalbuminemia, microscopic hematuria, proteinuria, creatinine, urea nitrogen, LDH, ESR, and abnormal elevated CRP were selected. No collinearity existed among them (variance inflation factor < 3, Table 3).

**Table 3:** Significant variables observed on single factor unconditional logistic regression and variance inflation factor diagnosis.

Variables	VIF
Age	1.218
SLE duration	1.143
Epilepsy	1.075
Fever	1.244
Serositis	1.208
Oral ulcer	1.082
Alopecia	1.075
Anemia	1.304
Urine protein	1.514
Urine RBC	1.464
24-h urine protein	1.335
Hypoproteinemia	1.535
Creatinine	1.068
Urea nitrogen	1.092
LDH	1.207
ESR	1.156
Elevated CRP	1.170
IgM	1.023
IgA	1.059
SLEDAI	1.576
Pulmonary hypertension	1.091
ILD	1.041
Pathogen infection	1.170

VIF, variance inflation factor; SLE, systemic lupus erythematosus; RBC, red blood cell; LDH, lactate dehydrogenase; ESR, erythrocyte sedimentation rate; CRP, C-reactive protein; Ig, immunoglobulin; ILD, interstitial lung disease; SLEDAI, systemic lupus erythematosus disease activity index; elevated CRP, CRP > 8 mg/L.

After multivariate unconditional logistic regression was conducted, shorter disease duration, younger age, moderate and severe anemia, proteinuria, abnormally elevated serum creatinine, interstitial lung disease, and infection were considered to be statistically associated with DAH in SLE (Table 4).

**Table 4:** Significant variables for diffuse alveolar hemorrhage in patients with systemic lupus erythematosus observed in multivariate unconditional logistic regression.

Variables	Odds ratio	95% CI	*p*
Age (year)	0.867	(0.764, 0.984)	0.027
SLE duration (year)	0.972	(0.946, 0.998)	0.034
Anemia			< 0.001
Mild	2.246	(0.220, 22.917)	0.495
Moderate	25.949	(3.316, 203.065)	0.002
Severe	24.904	(2.675, 231.859)	0.005
Extreme severe	49.694	(0.405, 6095.812)	0.111
24-h urine protein	10.839	(1.351, 86.938)	0.025
Creatinine (μmmol/L)			< 0.001
< 40	1.517	(0.571, 4.035)	0.403
> 135	14.534	(5.012, 42.142)	< 0.001
ILD	6.569	(2.053, 21.021)	0.002
Pathogen infection	8.890	(3.580, 22.077)	< 0.001

CI, confidence interval; SLE, systemic lupus erythematosus; ILD, interstitial lung disease; Mild anemia, hemoglobin level(g/L)90–110; Moderate anemia, hemoglobin level(g/L)60–89; Severe anemia, hemoglobin level(g/L)30–59; Extreme severe anemia, hemoglobin level(g/L) < 30.

### ROC curve and AUC analyses

**Figure 1:**
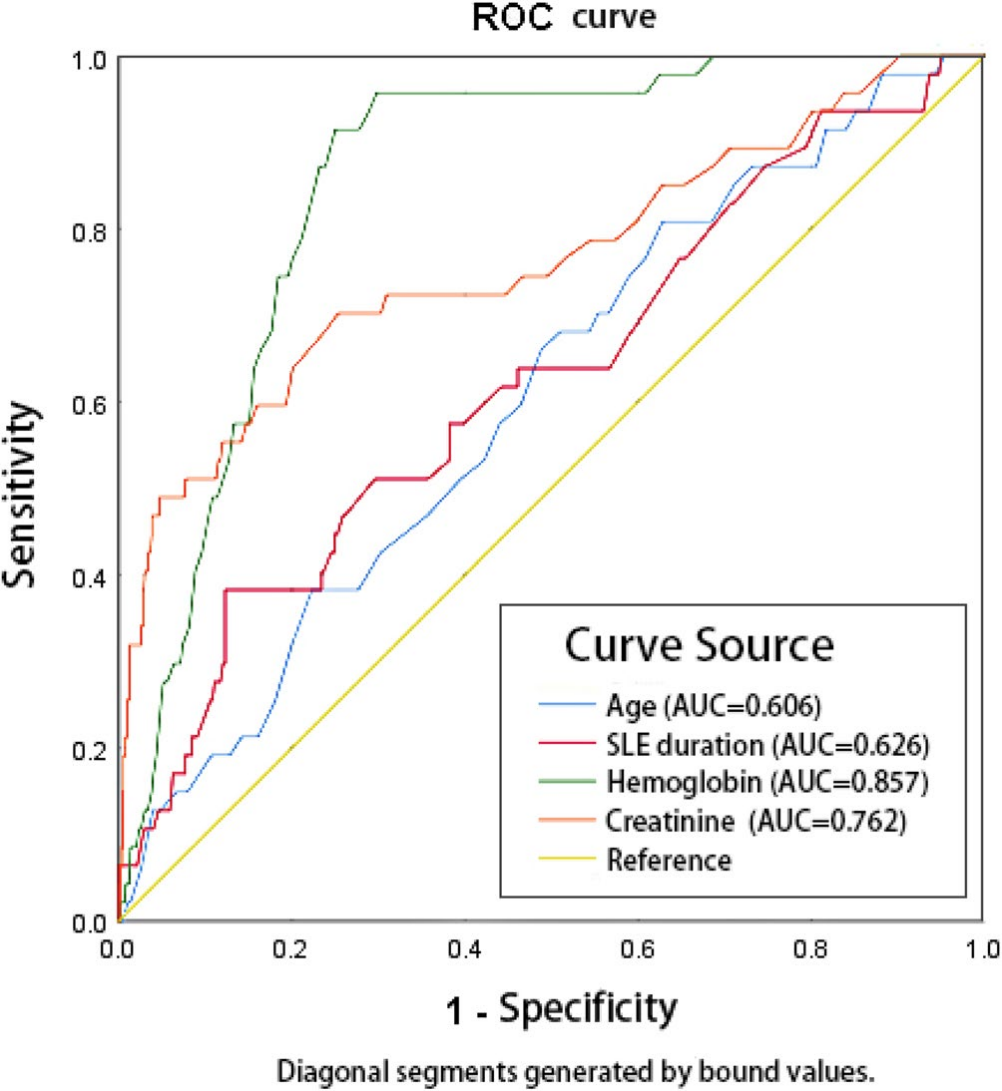
ROC curve analysis for risk factors of age, disease duration, levels of hemoglobin and creatinine. ROC, receiver operating characteristic; AUC, area under the curve; SLE, systemic lupus erythematosus.

ROC curve and AUC analyses were performed to assess the diagnostic efficiency of the above continuous variables, such as age, disease duration, and levels of hemoglobin and serum creatinine. The sensitivity and specificity for age were 80.9% and 37.2%, respectively, with AUC of 0.606. The sensitivity and specificity for disease duration were 38.3% and 87.7%, respectively, with AUC of 0.26. The sensitivity and specificity for hemoglobin were 91.5% and 75.0%, with AUC of 0.857. The sensitivity and specificity for creatinine were 70.2% and 74.7%, with AUC of 0.762 (Fig. 1).

## Discussion

DAH is a rare and potentially fatal complication of SLE. The DAH frequency in hospitalized patients with SLE varied from 0.2 to 5.4%^9,15,16^. In the present study, the frequency was 4.98%, which was within the previously reported range. Even though the mortality for SLE-associated DAH was previously reported to be as high as 70–90%^8^, it has been decreasing gradually over the past 20 years. The reduction in mortality may be due to the advances in immunology; further understanding of the pathogenesis of SLE; early recognition of DAH by means of thoracic HRCT scan; and advances in treatments, including rituximab^17–20^. The mortality rate of 29.41% was attributed to triple therapy (pulse intravenous methylprednisolone, cyclophosphamide, and plasmapheresis)^21^. The mortality rate of this complication in the present study was 42.55%. Though 15 patients (31.91%) with DAH received pulse intravenous methylprednisolone therapy, five of them died. Cyclophosphamide can produce cytotoxic effects by generating phosphamide mustard and reduce the production of antibodies when combined with glucocorticoids^22^. Similar to Ednalino et al.’s report^23^, the combination of glucocorticoids and cyclophosphamide was the most common treatment for patients with SLE-associated DAH in the present study. Meanwhile, plasmapheresis was used in 23.40% of patients with DAH in the present study. The mechanism of plasmapheresis may ameliorate pulmonary capillary inflammation by removing circulating immune complexes and autoantibodies. Jones et al. studied the long-term response to plasmapheresis in eight patients with SLE. The levels of immune complexes and autoantibodies in three patients who received glucocorticoids therapy or the combination of glucocorticoids and cytotoxic drugs during plasmapheresis significantly reduced compared with those before treatment, and they maintained a long-term downward trend. However, the other five patients who did not receive cytotoxic drugs and other treatments after plasmapheresis experienced a temporary decline in the levels of immune complex and autoantibodies, and then the levels quickly rebounded^24^. Selective removal of antibodies from dogs, followed by appropriate combination of cytotoxic drugs, may lead to a reduction in the synthesis of some specific antibodies. This finding suggested that for some time, after antibody removal, B cell clones underwent a rapid proliferation phase, during which they were particularly sensitive to destruction by cytotoxic drugs^25^. Rituximab recognizes CD20 in B cells, and when the antibody binds to the receptor, it triggers B cell death. B-cell depletion therapy has been successfully used in the treatment of recurrent catastrophic SLE^26–28^. Rituximab may be used as an effective means to prevent the rebound of autoantibody levels after plasmapheresis. In the present work, five patients received rituximab as a B-cell targeted therapy, and four patients survived. Besides B cell depletion, anifrolumab, a kind of type I interferon receptor antibody, has been applied to treat patients with moderate to severe SLE^29^. Janus kinase (JAK) is involved in diverse immune abnormalities. JAK inhibitors are a potential novel therapy for SLE^30,31^.

Diffuse alveolar hemorrhage could be the initial manifestation of SLE^32^. The present work showed that DAH was the initial symptom in 17 patients (36%), and 63.8% of patients experienced alveolar hemorrhagic attack within the first year of SLE. However, DAH occurred in 11 (23.40%) patients 3 years after the onset of SLE, and the longest onset was 14 years after the diagnosis of SLE. This finding was similar with that of Martínez-Martínez^19^. The present work showed that shorter disease duration and younger age were independent risk factors for DAH in patients with SLE.

Consistent with the report of Andrade^15^, anemia was found to be an independent risk factor for DAH in the present study. Loss of hemoglobin possessed the highest predictive value of DAH based on the biggest AUC. Not every DAH could lead to obvious hemoptysis even though evidence of infiltration images on pulmonary HRCT exists^23^.

According to the findings of Ednalino C^24^, lupus nephritis was fundamentally associated factor for DAH in SLE. The present study showed that abnormally elevated levels of proteinuria and serum creatinine were associated with DAH in patients with SLE.

Due to the disturbance of the immune system in patients with SLE and the administration of glucocorticoids and immunosuppressants, patients with SLE are vulnerable to infection. When DAH occurs, the blood accumulated in the alveolar space provides good cultural environment for microorganisms. The inflammatory response induced by infection or auto-immunity could damage the lung tissue. This indiscriminate attack destroys the integrity of small blood vessels in the lungs, creating conditions for the occurrence of DAH. Therefore, empiric antibiotic therapy could be initiated for SLE-associated DAH even without definite manifestation of respiratory infection^33^. The present study showed that 39 patients (82.98%) with DAH were complicated with infection, and those patients with infection had an 8.89 times greater risk of DAH than those without infection, which was another factor that was worth regarded as a probable etiology for the development of DAH^34^.

The ILD frequency in patients with SLE was reported to be 11.1%^13^. Those patients with ILD in the present study had a 6.569-fold greater risk of developing DAH than those without ILD. Therefore, clinicians are recommended to closely monitor patients with SLE complicated with ILD, periodically review chest CT, and be alert to DAH when new diffuse infiltrating image is obtained. SLEDAI (more than 10) and pulmonary hypertension were reported to be considered as independent risk factors of SLE complicated with DAH^17^. However, in the present study, even though statistical differences were observed in terms of SLEDAI-2K score and pulmonary hypertension between the DAH and non-DAH groups, neither one was an independent risk factor for patients with SLE complicated with DAH (Table 5). The SLEDAI score system has a limitation because it neglects to evaluate some potentially severe manifestations, such as interstitial lung disease, pulmonary hypertension, and hemolytic anemia. This study has some limitations. The data that retrospectively originated from a single center may not be a well representative of the general Chinese population. Moreover, bronchoalveolar lavage fluid testing was not performed in every patient with DAH in this study. Hence, further prospective studies with long-term follow-up in multiple centers are needed.

**Table 5:** Studies on risk factors of patients with SLE-DAH.

References	Country	Analytical method	DAH cases	Control cases	Viewpoint
Kwok et al.^17^	Korea	A multi-hospital retrospective analysis	21	83 cases matched from 1500	Neuropsychiatric lupus and high SLE disease activity index scores (SLEDAI > 10) are independent risk factors in the development of DAH
Kazzaz et al.^35^	America	A case–control cohort study	22	66 cases matched from about 1000	History of thrombocytopenia and low C3 are maintained as independent risk factors
Kim et al.^12^	Korea	Retrospective cohort	24	23	It is important to promptly evaluate the DAH when patients have low levels of hemoglobin or C4, and symptoms of hypoxia
Sun et al.^16^	China	A retrospective nested case–control analysis in a single-center	94	188 cases matched from 4650	LN, anti-SSA positivity, thrombocytopenia and elevated CRP are independent risk factors of DAH in lupus patients
Xu et al.^36^	China	Meta analysis	–	–	Neuropsychiatric involvement, nephritis, higher SLEDAI-2 K scores, and low levels of C3, C4, platelets, and hemoglobin may be correlated with the development of DAH in SLE patients
Xu et al. (this study)	China	A single center retrospective study	47	896	Shorter disease duration, younger age, moderate or severe anemia, abnormally elevated levels of urine protein and serum creatinine, interstitial lung disease, and infection are risk factors for the occurrence of DAH in patients with SLE. Moderate or severe anemia is highly suggestive of DAH

SLE, systemic lupus erythematosus; SLE-DAH, systemic lupus erythematosus-diffuse alveolar hemorrhage; SLEDAI, systemic lupus erythematosus disease activity index; LN, lupus nephritis; CRP, C-reactive protein; Moderate anemia, hemoglobin level (g/L) 60–89; Severe anemia, hemoglobin level (g/L) 30–59.

## Conclusions

This study shows that the risk factors of DAH in Chinese patients with SLE are short-term disease duration, young age, moderate or severe anemia, renal function impairment (abnormally elevated levels of urine protein and serum creatinine), interstitial lung disease, and infection. Moderate or severe anemia is highly suggestive of DAH.

## Data Availability

The data supporting the findings of this study are available from the corresponding authors upon reasonable request. The data are not publicly available due to privacy or ethical restrictions.
